# Supplementary material for: Metagenome analysis from the sediment of river Ganga and Yamuna: In search of beneficial microbiome
Source: PLoS One. 2020 Oct 6;15(10):e0239594. doi: 10.1371/journal.pone.0239594 (PMC7537857; doi:10.1371/journal.pone.0239594)
Supplement: S1 Table — (DOCX) [file pone.0239594.s001.docx]

**S1 Table:** Health benefit of identified bacteria and their proposed mechanism of action

| **Sl. No.** | **Probiotics species** | **Important health benefits** | **Proposed mechanism of action** | **References** |
| --- | --- | --- | --- | --- |
| 1 | *Vibrio mediterranei* | Antimicrobial activity | Produce a bacteriocin-like substance inhibiting growth of *V. parahaemolyticus* spp. and other closely related bacteria. | Carraturo et al. 2006 |
| 2 | *Vibrio fluvialis* | Improve disease resistance | Enhanced pathogen-resistant and lysozyme activity in the fish | Irianto and Austin (2002a, b) |
| 3 | *Vibrio harveyi* | Improve immune response | Significant changes in gill cells metabolism and immune response | Pichon et al.2013 |
| 4 | *Vibrio alginolyticus* | Improve disease resistance | Decrease of mortality of juveniles challenged with *A. salmonicida, V.anguillarum* and *V. ordalii* | Austin et al.1995 |
|  |  |  | *Vibrio alginolyticus* (NCIMB 1339) and *Vibrio gazogenes* (NCIMB 2250) showed antagonistic activity towards a panel of shrimp pathogenic *Vibrios*. In the case of *V. alginolyticus*, this activity depended on the presence of live bacteria while in *V. gazogenes* both live and dead bacteria showed anti-*Vibrio* activity. | Thompson et al., 2010 |
| 5 | *Bacillus Clausii* | Improve immune response | Improved growth performance and immune responses of *E. coioides* | Yun-Zhang et al. 2010 |
| 6 | *Bacillus circulans* | Improved growth performance | Better growth and survival of rohu spawn | Ghosh et al. 2004 |
| 7 | *Bacillus subtilis* | Antimicrobial activity | Increased the survival rate of prawns against *Aeromonas hydrophila* | Mehran & Masoumeh, 2012 |
| 8 | *Bacillus coagulans* | Improved growth performance and immune responses | Protective effect in Salmonella Enteritidis infected broilers | Zhen et at., 2018 |
| 9 | *Bacillus cereus* | Improve growth and immunity | B. cereus at a concentration of 0.4%/100 g feed was efficient in stimulating the growth and immunity in shrimp. | NavinChandran et al., 2014 |
| 10 | *Bacillus megaterium* | Enhence immunity | *Pontibacter* spp. and *Bacillus megaterium* included in feeds enhanced both assimilating capacity and immunological responses in *Labeo rohita*. | Sumathi et al., 2014 |
| 11 | *Bacillus mycoides* | Improve health | *Bacillus mycoides* (A10) and PM4 are the most beneficial dietary probiotics for marron health. | Ambas et al., 2013 |
| 12 | *Bacillus pumilus* | Improve disease resistance | Effective application of *B. pumilus* as a probiotic for streptococcosis resistance in both laboratory and field culture conditions. | Srisapoome and Areechon 2017 |
| 13 | *Bacillus Licheniformis* | Improve disease resistance | *B. licheniformis* Dahb1 can be applied in diet at 107 cfu g-1 to improve healthy status and resistance against *A. hydrophila* in tilapia farming. | Gobi et al., 2018 |
| 14 | *Lactobacillus Curvatus* | Lowered the cholesterol level | *Lact. curvatus* DN317 lowered the cholesterol level | Zommiti et al., 2017. |
| 15 | *Lactobacillus brevis* | Detoxification of aflatoxin | *L. brevis* exhibited the highest reduction of aflatoxin B1 production by *A. flavus* and *A. parasiticus*, 96.31 and 90.43%, respectively. | Gomaa et al., 2017 |
| 16 | *Lactobacillus helveticus* | Attenuates Experimental Autoimmune Encephalomyelitis | LH2171 significantly inhibited IL-6 production in vitro from both DC2.4 and RAW264.7 cells, model cell lines of antigen-presenting cells. These findings suggest that LH2171 might down-regulate IL-6 production and the subsequent Th17 differentiation and spinal cord infiltration, consequently alleviating EAE symptoms. | Yamashita et al., 2018 |
| 17 | *Lactobacillus buchneri* | Lowered the cholesterol level | *Lactobacillus buchneri* P2 with efficient cholesterol-reducing ability was isolated to provide species diversity of lactobacilli for functional dairy products. | Zeng et al., 2010 |
| 18 | *Lactobacillus Gasseri* | Reduce inflammation | Moreover, the two selected strains (*L. gasseri* 4M13and 5R13) substantially inhibited the release of inflammatory mediators such as TNF-α, IL-6, IL-1β, and IL-10 stimulated the treatment of RAW 264.7 macrophages with LPS. In addition, whole genome sequencing and comparative genomic analysis of 4B15 and 4M13 indicated them as novel genomic strains. | Oh et al., 2018 |
| 19 | *Lactobacillus paracasei* | Improves the control of secondary experimental meningococcal infection in flu-infected mice | Oral consumption of *L. paracasei* CNCM I-1518 reduced the weight loss of infected mice and lowered the bioluminescent signal of infecting meningococci. This improvement was associated with higher recruitment of inflammatory myeloid cells, such as interstitial monocytes and dendritic cells, to the lungs. | Belkacem et al., 2018 |
| 20 | *Lactobacillus crispatus* | Antimicrobial activity | The seven isolates showed good inhibitory activity against tested pathogens, high antioxidant activity (32.29% to 73.36%), and good ability to reduce cholesterol (22.55% to 75.15%). Thus, the seven tested strains have value as probiotics. Probiotic Properties of Exopolysaccharide-Producing *Lactobacillus* Strains Isolated from Tempoyak. | Khalil et al., 2018 |
| 21 | *Lactobacillus rhamnosus* | The immunomodulatory effects of probiotic formulation | Probiotic formulation induced a significant increase in anti-inflammatory cytokine interleukin-10 (IL-10) production and was able to decrease the secretion of the major proinflammatory cytokines IL-1β and IL-6 by 70% and 80%, respectively. | Sichetti et al., 2018 |
| 22 | *Lactobacillus acidophilus* | Reduce inflammation in inflammatory bowel disease (IBD) | L. acidophilus treatment directly induced T regulatory (Treg) cells and the production of IL-10, whereas the production of IL-17 was suppressed in splenocytes. | Park et al., 2018 |
| 23 | *Lactobacillus plantarum* | Relieving colitis by gut microbiota, immune, and anti-oxidative stress | *L. plantarum* ZDY2013 and *B. bifidum* WBIN03 remit Ulcerative colitis (UC) through modification of gut microbiota to regulate oxidative stress and inflammatory mediators. | Wang et al., 2018 |
| 24 | *Lactobacillus casei* | Anti stress | Seven *L. casei* and six *L. fermentum* strains produced β-galactosidase enzymes, and ten strains survived well the simulation of the GIT stressful conditions evaluated in vitro. | de Souza et al., 2018 |
| 25 | *Lactobacillus delbrueckii* | Anti-inflammatory | Strain-specific anti-inflammatory property of native cultures may be useful to alleviate inflammatory conditions and develop a target based probiotic. | Catherine et al., 2018 |
| 26 | *Lactobacillus salivarius* | Anti-inflammatory, attenuate skin inflammation in mice. | Treatment with the two Lactobacillus strains(*L. salivarius* LA307 and *L. rhamnosus* LA305) induced a decrease in the serum levels of pro-inflammatory cytokines IL-1β, IL-6, TNF-α, IL-17, IL-22 and at the opposite an increase in the production of the anti-inflammatory cytokine IL-10 and also of IL-4. | Holowacz et al., 2018 |
| 27 | *Lactobacillus johnsonii* | Anti-giardial activity | Bile-Salt-Hydrolase (BSH)-like activities from the probiotic strain of Lactobacillus johnsonii La1 may contribute to the anti-giardial activity displayed by this strain. | Allain et al., 2018 |
| 28 | *Lactobacillus fermentum* | Antibacterial activity | *L. fermentum* UCO-979C strongly inhibited the colonization of *H. pylori* decreasing up to 87% of the colonisation in the antrum by the pathogen, suggesting that this probiotic strain has a strong probiotic activity against *H. pylori* in the most valuable animal model for in vivo assays nowadays. | Merino et al., 2018 |
| 29 | *Lactobacillus reuteri* | Reduce gut inflammation | KLD is a mixture of krill oil (KO), probiotic *Lactobacillus reuteri* (LR), and vitamin D (VitD3). KLD has significant effects on the intestinal mucosa, strongly decreasing inflammation, increasing epithelial restitution and reducing pathogenicity of harmful commensal bacteria. | Costanzo et al., 2018 |
| 30 | *Bifidobacteriumanimalis* | Ameliorate neuroinflammation | The results also revealed that treatment with combination of both strains (*L*. *plantarum* and *B. animalis*) enhanced the population of CD4+CD25+Foxp3+-expressing T-cells in the lymph nodes and the spleen. *Bifidobacterium animalis* in combination with human origin of Lactobacillus plantarum ameliorate neuroinflammation in experimental model of multiple sclerosis by altering CD4+ T cell subset balance. | Salehipour et al., 2017 |
| 31 | *Bifidobacteriumbifidum* | Beneficial effects on gastrointestinal discomfort | Daily consumption of YIT10347 (*Bifidobacterium bifidum* YIT 10347) fermented milk exerts beneficial effects on gastrointestinal discomfort and symptoms such as postprandial discomfort and epigastric pain in healthy adults. | Gomi et al., 2018 |
| 32 | *Bifidobacteriumlongum* | Anti-obese effects | The mixture of fermented ginseng and B. longum BORI and L. paracasei CH88 could have anti-obese effects and suppress lipid deposit in the liver and adipose tissues. | Kang D, Li Z, Ji GE. Et al., 2018 |
| 33 | *Bifidobacteriumbreve* | Anti-inflammatory | Gene profiling analysis revealed that the consumption of B. breve A1 suppressed the hippocampal expressions of inflammation and immune-reactive genes that are induced by amyloid-β. Together, these findings suggest that B. breve A1 has therapeutic potential for preventing cognitive impairment in AD. | Kobayashi et al., 2017 |
| 34 | *Bifidobacteriumadolescentis* | Ameliorates high-fat diet-induced colitis | *Bifidobacterium adolescentis* IM38 can inhibit HFD-induced LPS production in gut microbiota through the regulation of Proteobacteria to Bacteroidetes ratio and NF-κB activation in the colon, which ultimately attenuates colitis. | Lim and Kim, 2017 |
| 35 | *Shewanellacolwelliana* | Improve innate immunity and disease resistance | Potential for *S. colwelliana* WA64 and *S. olleyana* WA65 to improve innate immunity and disease resistance in *H. discus hannai* and survival of juvenile abalone challenged with *Vibrio harveyi* have been studied. | Jiang et al., 2013 |
| 36 | *Shewanellaputrefaciens* | Improve growth and immunity | Application of *Shewanella putrefaciens* Pdp11 in the first life stages of *S*. *senegalensis* as an effective tool with the clear potential to benefit sole aquaculture. | Jurado et al., 2018 |
| 37 | *Pseudomonas fluorescens* | Anti-microbial | Results of this study suggest that a major part of the antagonistic property exhibited by strain *Pseudomonas fluorescens* AH2 is caused by the ability of siderophores in the supernatant to efficiently chelate iron, which results in instant iron deprivation of the pathogen *V. anguillarum* and complete growth arrest. | Holmstrøm and Gram, 2003 |
| 38 | *Pediococcuspentosaceus* | Anti-inflammation and cancer | Conclusively, novel probiotic strain *Pediococcus pentosaceus* strain GS4 exhibited safe and beneficial effects against the toxicity threats posed by azoxymethane. Thus, GS4 could be considered as a potential food supplement/additive for therapeutic purposes in gastrointestinal disorders related to inflammation and cancer. | Dubey et al., 2015 |
| 39 | *Pediococcusacidilactici* | Improve reproductive performance | Combined diet (*Pediococcus acidilactici* 0.2% and nucleotide) had the highest percentage and duration of sperm motility, absolute fecundity, and fertilization success as compared to other diets (P < 0.05) in Goldfish (*Carassius auratus*). | Mehdinejad et al., 2018 |
| 40 | *Enterococcus durans* | Supress colitis | *E. durans* TN-3 exerted an inhibitory effect on the development of dextran sulfate sodium (DSS) colitis. This action might be mediated by the induction of Treg cells and the restoration of the diversity of the gut microbiota. | Kanda et al., 2016 |
| 41 | *Enterococcus faecium* | Decreases the signs and symptoms of atopy | *Bifidobacterium animalis* subsp. lactis BB12 and *Enterococcus faecium* L3 significantly reduced (p<0.001) rhinitis, watery eyes and cough/bronchospasm. However, reduced efficacy was observed when the mixture was given during the 3 months of atopy. | Di Pierro et al., 2018 |
| 42 | *Streptococcus thermophilus* | Prevention and control of hypertension. | This study investigated the microbial viability, proteolysis and angiotensin-converting enzyme (ACE) inhibitory activity of a fermented dairy beverage produced with goat whey powder and a probiotic culture of L. casei BGP93 co-cultured with Streptococcus thermophilus TA-40. | Pereira et al., 2017 |
| 43 | *Leuconostocmesenteroides* | Cancer prevention and therapy | *Leuconostoc mesenteroides* derived anticancer pharmaceuticals hinder inflammation and cell survival in colon cancer cells by modulating NF-κB/AKT/PTEN/MAPK pathways. | Zununi et al., 2017 |
| 44 | *Aeromonashydrophila* | Improve growth | Stereology and computer assisted three-dimensional reconstruction as tools to study probiotic effects of *Aeromonas hydrophila* on the digestive tract of germ-free *Artemia franciscana nauplii*. Slight increment in the growth of the digestive tract of *A. Franciscana nauplii* exerted by probiotic bacteria could be detected using stereology and three-dimensional reconstruction. | Gunasekara et al., 2011 |
| 45 | *Micrococcus luteus* | Enhanced the fish growth and health | *M. luteus* enhanced the fish growth and health. It is recommended to use *M*. *luteus* as a probiotic in vivo | Abd El-Rhman et al., 2009 |
| 46 | *Paenibacilluspolymyxa* | Improve growth, feed utilization, non-specific immune responses | The challenge test showed dietary supplementation of *B. coagulans*, *B*. *licheniformis* and *P. polymyxa* significantly (p < 0.05) enhanced the resistance of fish fry against bacterial challenge. These results collectively suggests that *P*. *polymyxa* is a potential probiotic species and can be used in aquaculture to improve growth, feed utilization, non-specific immune responses and disease resistance of fry common carp, *C. carpio*. | Gupta et al., 2014 |
| 47 | *Lactococcuslactis* | Prevent and treat periodontitis and halitosis | L. lactis suspension had a neutralizing effect on the volatile sulfur compounds (VSCs) produced by periodontopathogens, and UV-killed *L. lactis* inhibited the production of IL-6 and TNF-α induced by the LPS. These results suggest that *L*. *lactis* may be a useful probiotic to prevent and treat periodontitis and halitosis. | Shin et al., 2018 |
| 48 | *Carnobacteriumdivergens* | Reduce the extent of Campylobacter spp. invasion | Results of our study indicate that probiotic (Lavipan composed of *Lactococcuslactis*, *Carnobacteriumdivergens*, *Lactobacillus casei*, *Lactobacillus plantarum* and *Saccharomyces cerevisae* to the feed) added to a feed for broiler chickens was capable to reduce the extent of Campylobacter spp. invasion in the gastrointestinal tract of birds and, resultantly, to diminish contamination level in bird environment, which eventually contributed to the improved hygienic parameters of analyzed poultry carcasses. | Smialek et al., 2018 |
| 49 | *Carnobacteriummaltaromaticum* | Enhanced the cellular and humoral immune responses | The cultures enhanced the cellular and humoral immune responses. Specifically, fish fed with *Carnobacterium maltaromaticum* B26 demonstrated significantly increased phagocytic activity of the head kidney macrophages, whereas the use of *Carnobacterium divergens* B33 led to significant increases in respiratory burst and serum lysozyme activity. Also, the gut mucosal lysozyme activity for fish fed with both cultures was statistically higher than the controls. | Kim and Austin, 2006 |
| 50 | *Roseobacterlitoralis* | Antibacterial Compounds | Production of Antibacterial Compounds and Biofilm Formation by *Roseobacter* Species Are Influenced by Culture Conditions | Bruhn et al., 2007 |
| 51 | *Roseobacterdenitrificans* | Peroxidase activity and involvement in the oxidative stress response of roseobacterdenitrificans truncated hemoglobin | Genomic organization and gene expression profiles imply possible functions for detoxification of reactive oxygen and nitrogen species in vivo. Altogether, Rd. trHb exhibits some distinctive features and appears equipped to help the bacterium to cope with reactive oxygen/nitrogen species and/or to operate redox biochemistry. | Wang et al., 2015 |
| 52 | *RoseobacterlitoralisOch 149* | Serine racemase is an enzyme which generates D-serine from L-serine. D-serine acts as a neuronal signaling molecule by activating NMDA receptors in the brain. | Distribution of eukaryotic serine racemases in the bacterial domain and characterization of a representative protein in *Roseobacter litoralis* Och 149. The catalytic efficiency for L-serine racemization of RiSR (kcat/Km=3.14 min (-1) mM (-1)) was 34-fold higher than that of l-serine dehydration. | Kubota et al., 2016 |
| 53 | *RoseobacterdenitrificansOCh 114* | No probiotics report found | The Complete Genome Sequence of *Roseobacter denitrificans* Reveals a Mixotrophic rather than photosynthetic metabolism | Swingley et al., 2007 |
| 54 | *Vagococcusteuberi* | Lactic acid bacteria belonging to the genus*Vagococcus* | *Vagococcusteuberi* sp. nov., isolated from the Malian artisanal sour milk fènè. | Wullschleger et al., 2018 |
| 55 | *Oenococcusoeni* | Lowered colonic injury and alleviated colitis symptoms | The 'natural' tolerance towards acid, ethanol, and phenolic compounds of *O. oeni* strains combined with a measureable immunomodulatory potential, suggest a possible use of selected strains isolated from wine as live probiotics. | Foligné et al., 2010 |
| 56 | *Oenococcuskitaharae* | Bacteriocins production | However *O. kitaharae* appears to have adapted to a growth environment in which biological competition provides a significant selective pressure by accumulating biological defense molecules, such as bacteriocins and restriction-modification systems, throughout its genome. | Borneman et al., 2012 |
| 57 | *Oenococcusalcoholitolerans* | Improve health | *Oenococcusalcoholi tolerans* sp. nov., a lactic acid bacteria isolated from cachaça and ethanol fermentation processes. The 16S rRNA gene sequence similarity against the type strains of the other two species of the genus was below 94.76 % (*Oenococcuski taharae*) and 94.62 % (*Oenococcusoeni*). Distinctive phenotypic characteristics are the ability to metabolize sucrose but not trehalose. | Badotti et al., 2014. |
| 58 | *Pediococcusethanolidurans* | Cellular antioxidant and bile salt hydrolase (BSH) activities | Strains were further evaluated for hydroxyl radical scavenging activity, reducing power, and ferrous ion chelating activity exerted by both viable intact cells and/or intracellular cell-free extracts. | Xu et al., 2016 |
| 59 | *Aeromonasveronii* | Regulation of immunity | Modulation of intestinal environment by the probiotics could impact the abundance of cellulose-degrading bacteria (e.g., *Citrobacter* genus). Probiotics-induced alteration of microbiota may improve immunity and potentially lower the risk of disease outbreaks during cultivation stage of grass carp. | Hao et al., 2017 |
| 60 | *Enterococcus faecalis* | Enhance immunity | It was suggested that the immune response is enhanced due to antioxidative activity caused by the *Enterococcus faecalis*-2001 and anti-tumor activity by NK cells and TNF-α. | Gu et al., 2017 |
| 61 | *Enterococcus malodoratus* | Antimicrobial activity | The positive influence was followed in number of *Lactobacilli* and *Enterococci* in the experimental groups. | Kačániová et al., 2012 |
| 62 | *Enterococcus raffinosus* | Prevention of bacterial infections | exhibited strong antibacterial activities against all pathogens including *Aeromonas hydrophila* | Sahoo et al., 2015 |
| 63 | *Enterococcus hirae* | Antioxidant potentiality | Ability for the production of lipase and Bsh enzyme. It was also able to survive under simulated gastrointestinal conditions with the inhibition ability of various pathogens. The antioxidant potentiality with the cell surface hydrophobicity and cell aggregation ability confirms its potentiality as a potent probiotic. | Adnan et al., 2017 |
| 64 | *Enterococcus mundtii* | Antimicrobial activity | Larvae exposed to the *Enterococcus mundtii* showed increased survival following infection with *B. thuringiensis* | Grau et al., 2017 |
| 65 | *Pseudomonas chlororaphis* | Antimicrobial activity | Inoculation of juvenile perch with P. chlororaphis strain JF3835 prior to infection with A. sobria caused a reduction in *A. sobria* associated mortalities. | Gobeli et al., 2009 |
| 66 | *Pseudomonas stutzeri* | Antimicrobial activity | Candida utilis and Pseudomonas stutzeri are fairly adherent and play an important role in the enhancement of the protection of Artemia culture against pathogens. | Abdelkarim et al., 2010 |
| 67 | *Pseudomonas synxantha* | Antimicrobial activity | The prawns exposed to the combined probiotics were healthier than those exposed to the individual probiotics. P. aeruginosa was more effective for improving prawn health than P. synxantha. The probiotic-fed prawns were not influenced by Vibrio harveyi at 10(3) CFU ml(-1) for 36 h of challenge. | Van et al., 2009 |
| 68 | *Shewanellaxiamenensis* | Antimicrobial activity | Members of the genera *Aeromonas* and *Shewanella* appeared to communicate with each other by using the QS system to some extent when the concentration of AHL reaches a certain threshold. It is therefore suggested that bacteria with the ability to disrupt AHL secretion in intestinal environments are potential candidates for probionts for preventing opportunistic infections in freshwater fish such as goldfish. | Sugita et al., 2017 |
| 69 | *Streptococcus salivarius* | Antimicrobial activity | It is found that BglB, CshA, GtfH and LiaR were specifically associated with bacterial auto-aggregation, whereas Asp1, Asp2, CwpB, CwpK, GtfE, GtfG, SecA2 and SrtA also contributed to adhesion to host cells and host-derived components, or to interactions with the human pathogen *Fusobacterium nucleatum*. | Couvigny et al., 2018 |
