# Supplementary material for: Metagenome analysis from the sediment of river Ganga and Yamuna: In search of beneficial microbiome
Source: PLoS One. 2020 Oct 6;15(10):e0239594. doi: 10.1371/journal.pone.0239594 (PMC7537857; doi:10.1371/journal.pone.0239594)
Supplement: S2 Table — (DOCX) [file pone.0239594.s002.docx]

**S2 Table.** Relative abundance of beneficial bacteria species identified from the nine sediment metagenome of river Ganga and Yamuna

| **Name of the Genus** | **Name of the species** | **Reference paper** |
| --- | --- | --- |
|  | ***L. curvatus*** | - Zommiti, M., Connil, N., Hamida, J.B. and Ferchichi, M., 2017. Probiotic characteristics of *Lactobacillus curvatus* DN317, a strain isolated from chicken ceca. Probiotics and antimicrobial proteins, 9(4), 415-424. - Jung, S., Lee, Y.J., Kim, M., Kim, M., Kwak, J.H., Lee, J.W., Ahn, Y.T., Sim, J.H. and Lee, J.H., 2015. Supplementation with two probiotic strains, *Lactobacillus curvatus* HY7601 and *Lactobacillus plantarum* KY1032, reduced body adiposity and Lp-PLA2 activity in overweight subjects. Journal of Functional Foods, 19, 744-752. |
| ***Lactobacillus*** | ***L. brevis*** | - Kariyawasam, K.M.G.M.M., Yang, S.J., Lee, N.K. and Paik, H.D., 2020. Probiotic Properties of *Lactobacillus brevis* KU200019 and Synergistic Activity with Fructooligosaccharides in Antagonistic Activity against Foodborne Pathogens. Food Science of Animal Resources, 40(2), p.297. - Fang, F., Xu, J., Li, Q., Xia, X. and Du, G., 2018. Characterization of a *Lactobacillus brevis* strain with potential oral probiotic properties. BMC microbiology, 18(1), p.221. - Abdelazez, A., Abdelmotaal, H., Evivie, S.E., Melak, S., Jia, F.F., Khoso, M.H., Zhu, Z.T., Zhang, L.J., Sami, R. and Meng, X.C., 2018. Screening potential probiotic characteristics of *Lactobacillus brevis* strains in vitro and intervention effect on type I diabetes in vivo. BioMed research international, 2018. |
|  | ***L. casei*** | - Sutula, J., Coulthwaite, L.A., Thomas, L.V. and Verran, J., 2013. The effect of a commercial probiotic drink containing *Lactobacillus casei* strain Shirota on oral health in healthy dentate people. Microbial ecology in health and disease, 24(1), p.21003. - Hill, D., Sugrue, I., Tobin, C., Hill, C., Stanton, C. and Ross, R.P., 2018. The *Lactobacillus casei* group: history and health related applications. Frontiers in microbiology, 9, p.2107. - Marinaki, E., Kandylis, P., Dimitrellou, D., Zakynthinos, G. and Varzakas, T., 2016. Probiotic yogurt production with *Lactobacillus casei* and prebiotics. Current Research in Nutrition and Food Science Journal, 4(Special Issue Nutrition in Conference October 2016), 48-53. |
|  | ***L. acidophilus*** | - Kos, B.V.Z.E., Šušković, J., Vuković, S., Šimpraga, M., Frece, J. and Matošić, S., 2003. Adhesion and aggregation ability of probiotic strain *Lactobacillus acidophilus* M92. Journal of applied microbiology, 94(6), 981-987. - Kailasapathy, K. and Chin, J., 2000. Survival and therapeutic potential of probiotic organisms with reference to *Lactobacillus acidophilus* and Bifidobacterium spp. Immunology and cell biology, 78(1), 80-88. - Yadav, H., Jain, S. and Sinha, P.R., 2007. Antidiabetic effect of probiotic dahi containing *Lactobacillus acidophilus* and *Lactobacillus casei* in high fructose fed rats. Nutrition, 23(1), 62-68. |
|  | ***L. buchneri*** | - Zeng, X.Q., Pan, D.D. and Guo, Y.X., 2010. The probiotic properties of *Lactobacillus buchneri* P2. Journal of applied microbiology, 108(6), 2059-2066. - Rabelo, C.H.S., Basso, F.C., Lara, E.C., Jorge, L.G.O., Härter, C.J., Mesquita, L.G., Silva, L.F.P. and Reis, R.A., 2018. Effects of *Lactobacillus buchneri* as a silage inoculant and as a probiotic on feed intake, apparent digestibility and ruminal fermentation and microbiology in wethers fed low‐dry‐matter whole‐crop maize silage. Grass and forage science, 73(1), 67-77. - Annuk, H., Shchepetova, J., Kullisaar, T., Songisepp, E., Zilmer, M. and Mikelsaar, M., 2003. Characterization of intestinal lactobacilli as putative probiotic candidates. Journal of Applied Microbiology, 94(3), 403-412. - Cheon, M.J., Lim, S.M., Lee, N.K. and Paik, H.D., 2020. Probiotic Properties and Neuroprotective Effects of *Lactobacillus buchneri* KU200793 Isolated from Korean Fermented Foods. International Journal of Molecular Sciences, 21(4), p.1227. |
|  | ***L. crispatus*** | - Li, T., Liu, Z., Zhang, X., Chen, X. and Wang, S., 2019. Local probiotic *lactobacillus crispatus* and *lactobacillus delbrueckii* exhibit strong antifungal effects against vulvovaginal candidiasis in a rat model. Frontiers in Microbiology, 10, p.1033. - Stapleton, A.E., Au-Yeung, M., Hooton, T.M., Fredricks, D.N., Roberts, P.L., Czaja, C.A., Yarova-Yarovaya, Y., Fiedler, T., Cox, M. and Stamm, W.E., 2011. Randomized, placebo-controlled phase 2 trial of a *Lactobacillus crispatus* probiotic given intravaginally for prevention of recurrent urinary tract infection. Clinical infectious diseases, 52(10), 1212-1217. |
|  | ***L. delbrueckii*** | - Guglielmotti, D.M., Marcó, M.B., Golowczyc, M., Reinheimer, J.A. and Quiberoni, A.D.L., 2007. Probiotic potential of *Lactobacillus delbrueckii* strains and their phage resistant mutants. International Dairy Journal, 17(8), 916-925. - Otero, M.C., Espeche, M.C. and Nader-Macías, M.E., 2007. Optimization of the freeze-drying media and survival throughout storage of freeze-dried *Lactobacillus gasseri* and *Lactobacillus delbrueckii* subsp. delbrueckii for veterinarian probiotic applications. Process Biochemistry, 42(10), 1406-1411. - Boris, S., Jiménez‐Díaz, R., Caso, J.L. and Barbes, C., 2001. Partial characterization of a bacteriocin produced by *Lactobacillus delbrueckii* subsp. lactis UO004, an intestinal isolate with probiotic potential. Journal of applied microbiology, 91(2), 328-333. |
|  | ***L. fermentum*** | - Asan-Ozusaglam, M. and Gunyakti, A., 2019. *Lactobacillus fermentum* strains from human breast milk with probiotic properties and cholesterol-lowering effects. Food science and biotechnology, 28(2), 501-509. - Mikelsaar, M. and Zilmer, M., 2009. *Lactobacillus fermentum* ME-3–an antimicrobial and antioxidative probiotic. Microbial ecology in health and disease, 21(1), 1-27. - Ramos, C.L., Thorsen, L., Schwan, R.F. and Jespersen, L., 2013. Strain-specific probiotics properties of *Lactobacillus fermentum*, *Lactobacillus plantarum* and *Lactobacillus brevis* isolates from Brazilian food products. Food microbiology, 36(1), 22-29. - Bao, Y., Zhang, Y., Zhang, Y., Liu, Y., Wang, S., Dong, X., Wang, Y. and Zhang, H., 2010. Screening of potential probiotic properties of *Lactobacillus fermentum* isolated from traditional dairy products. Food Control, 21(5), 695-701. - Ghosh, K., Ray, M., Adak, A., Halder, S.K., Das, A., Jana, A., Parua, S., Vágvölgyi, C., Mohapatra, P.K.D., Pati, B.R. and Mondal, K.C., 2015. Role of probiotic *Lactobacillus fermentum* KKL1 in the preparation of a rice based fermented beverage. Bioresource technology, 188, 161-168. - Park, M.R., Ryu, S., Maburutse, B.E., Oh, N.S., Kim, S.H., Oh, S., Jeong, S.Y., Jeong, D.Y., Oh, S. and Kim, Y., 2018. Probiotic *Lactobacillus fermentum* strain JDFM216 stimulates the longevity and immune response of Caenorhabditis elegans through a nuclear hormone receptor. Scientific reports, 8(1), 1-10. |
|  | ***L. Gasseri*** | - Kadooka, Y., Sato, M., Imaizumi, K., Ogawa, A., Ikuyama, K., Akai, Y., Okano, M., Kagoshima, M. and Tsuchida, T., 2010. Regulation of abdominal adiposity by probiotics (*Lactobacillus gasseri* SBT2055) in adults with obese tendencies in a randomized controlled trial. European journal of clinical nutrition, 64(6), pp.636-643. - Selle, K. and Klaenhammer, T.R., 2013. Genomic and phenotypic evidence for probiotic influences of *Lactobacillus gasseri* on human health. FEMS microbiology reviews, 37(6), 915-935. - Ushiyama, A., Tanaka, K., Aiba, Y., Shiba, T., Takagi, A., Mine, T. and Koga, Y., 2003. *Lactobacillus gasseri* OLL2716 as a probiotic in clarithromycin‐resistant Helicobacter pylori infection. Journal of gastroenterology and hepatology, 18(8), 986-991. |
|  | ***L. helveticus*** | - Taverniti, V. and Guglielmetti, S., 2012. Health-promoting properties of *Lactobacillus helveticus*. Frontiers in microbiology, 3, p.392. - Ait-Belgnaoui, A., Payard, I., Rolland, C., Harkat, C., Braniste, V., Théodorou, V. and Tompkins, T.A., 2018. *Bifidobacterium longum* and *Lactobacillus helveticus* synergistically suppress stress-related visceral hypersensitivity through hypothalamic-pituitary-adrenal axis modulation. Journal of neurogastroenterology and motility, 24(1), p.138. - Fontana, A., Falasconi, I., Molinari, P., Treu, L., Basile, A., Vezzi, A., Campanaro, S. and Morelli, L., 2019. Genomic comparison of *Lactobacillus helveticus* strains highlights probiotic potentials. Frontiers in Microbiology, 10, p.1380. - Rong, J., Zheng, H., Liu, M., Hu, X., Wang, T., Zhang, X., Jin, F. and Wang, L., 2015. Probiotic and anti-inflammatory attributes of an isolate *Lactobacillus helveticus* NS8 from Mongolian fermented koumiss. BMC microbiology, 15(1), 1-11. |
|  | ***L. johnsonii*** | - Pridmore, R.D., Berger, B., Desiere, F., Vilanova, D., Barretto, C., Pittet, A.C., Zwahlen, M.C., Rouvet, M., Altermann, E., Barrangou, R. and Mollet, B., 2004. The genome sequence of the probiotic intestinal bacterium *Lactobacillus johnsonii* NCC 533. Proceedings of the National Academy of Sciences, 101(8), 2512-2517. - Van Gossum, A., Dewit, O., Louis, E., de Hertogh, G., Baert, F., Fontaine, F., DeVos, M., Enslen, M., Paintin, M. and Franchimont, D., 2007. Multicenter randomized-controlled clinical trial of probiotics (*Lactobacillus johnsonii*, LA1) on early endoscopic recurrence of Crohn's disease after ileo-caecal resection. Inflammatory bowel diseases, 13(2), 135-142. - Davoren, M.J., Liu, J., Castellanos, J., Rodríguez-Malavé, N.I. and Schiestl, R.H., 2019. A novel probiotic, *Lactobacillus johnsonii* 456, resists acid and can persist in the human gut beyond the initial ingestion period. Gut microbes, 10(4), 458-480. - Xin, J., Zeng, D., Wang, H., Sun, N., Zhao, Y., Dan, Y., Pan, K., Jing, B. and Ni, X., 2019. Probiotic *Lactobacillus johnsonii* BS15 promotes growth performance, intestinal immunity, and gut microbiota in piglets. Probiotics and Antimicrobial Proteins, 1-10. - Wang, H., Ni, X., Qing, X., Liu, L., Xin, J., Luo, M., Khalique, A., Dan, Y., Pan, K., Jing, B. and Zeng, D., 2018. Probiotic *Lactobacillus johnsonii* BS15 improves blood parameters related to immunity in broilers experimentally infected with subclinical necrotic enteritis. Frontiers in Microbiology, 9, p.49. - Wang, H., Ni, X., Qing, X., Zeng, D., Luo, M., Liu, L., Li, G., Pan, K. and Jing, B., 2017. Live probiotic *Lactobacillus johnsonii* BS15 promotes growth performance and lowers fat deposition by improving lipid metabolism, intestinal development, and gut microflora in broilers. Frontiers in Microbiology, 8, p.1073. |
|  | ***L. paracasei*** | - Phillips, M., Kailasapathy, K. and Tran, L., 2006. Viability of commercial probiotic cultures (*L. acidophilus, Bifidobacterium sp., L. casei, L. paracasei and L. rhamnosus*) in cheddar cheese. International journal of food microbiology, 108(2), 276-280. - Verdenelli, M.C., Ghelfi, F., Silvi, S., Orpianesi, C., Cecchini, C. and Cresci, A., 2009. Probiotic properties of *Lactobacillus rhamnosus* and *Lactobacillus paracasei* isolated from human faeces. European Journal of Nutrition, 48(6), 355-363. - Chuang, L.C., Huang, C.S., Ou-Yang, L.W. and Lin, S.Y., 2011. Probiotic *Lactobacillus paracasei* effect on cariogenic bacterial flora. Clinical oral investigations, 15(4), 471-476. - Buriti, F.C., da Rocha, J.S., Assis, E.G. and Saad, S.M., 2005. Probiotic potential of Minas fresh cheese prepared with the addition of *Lactobacillus paracasei*. LWT-Food Science and Technology, 38(2), 173-180. - Xu, Y., Tian, Y., Cao, Y., Li, J., Guo, H., Su, Y., Tian, Y., Wang, C., Wang, T. and Zhang, L., 2019. Probiotic Properties of *Lactobacillus paracasei* L1 and Its Growth Performance-Promotion in Chicken by Improving the Intestinal Microflora. Frontiers in physiology, 10, p.937. |
|  | ***L. plantarum*** | - Cebeci, A. and Gürakan, C., 2003. Properties of potential probiotic *Lactobacillus plantarum* strains. Food Microbiology, 20(5), 511-518. - De Vries, M.C., Vaughan, E.E., Kleerebezem, M. and de Vos, W.M., 2006. *Lactobacillus plantarum*-survival, functional and potential probiotic properties in the human intestinal tract. International Dairy Journal, 16(9), 1018-1028. - Zago, M., Fornasari, M.E., Carminati, D., Burns, P., Suàrez, V., Vinderola, G., Reinheimer, J. and Giraffa, G., 2011. Characterization and probiotic potential of *Lactobacillus plantarum* strains isolated from cheeses. Food Microbiology, 28(5), 1033-1040. - Arasu, M.V., Al-Dhabi, N.A., Ilavenil, S., Choi, K.C. and Srigopalram, S., 2016. In vitro importance of probiotic *Lactobacillus plantarum* related to medical field. Saudi journal of biological sciences, 23(1), S6-S10. - Belicová, A., Mikulášová, M. and Dušinský, R., 2013. Probiotic potential and safety properties of *Lactobacillus plantarum* from Slovak Bryndza cheese. BioMed research international, 2013. |
|  | ***L. reuteri*** | - Krasse, P., Carlsson, B., Dahl, C., Paulsson, A., Nilsson, A. and Sinkiewicz, G., 2006. Decreased gum bleeding and reduced gingivitis by the probiotic *Lactobacillus reuteri*. Swedish dental journal, 30(2), 55-60. - Jones, S.E. and Versalovic, J., 2009. Probiotic *Lactobacillus reuteri* biofilms produce antimicrobial and anti-inflammatory factors. BMC microbiology, 9(1), 1-9. |
|  | ***L. rhamnosus*** | - Salminen, M.K., Tynkkynen, S., Rautelin, H., Saxelin, M., Vaara, M., Ruutu, P., Sarna, S., Valtonen, V. and Järvinen, A., 2002. *Lactobacillus* bacteremia during a rapid increase in probiotic use of *Lactobacillus rhamnosus* GG in Finland. Clinical infectious diseases, 35(10), 1155-1160. - Nikoskelainen, S., Ouwehand, A.C., Bylund, G., Salminen, S. and Lilius, E.M., 2003. Immune enhancement in rainbow trout (*Oncorhynchus mykiss*) by potential probiotic bacteria (*Lactobacillus rhamnosus*). Fish & shellfish immunology, 15(5), 443-452. - De Groote, M.A., Frank, D.N., Dowell, E., Glode, M.P. and Pace, N.R., 2005. *Lactobacillus rhamnosus* GG bacteremia associated with probiotic use in a child with short gut syndrome. The Pediatric infectious disease journal, 24(3), 278-280. - Panigrahi, A., Kiron, V., Kobayashi, T., Puangkaew, J., Satoh, S. and Sugita, H., 2004. Immune responses in rainbow trout *Oncorhynchus mykiss* induced by a potential probiotic bacteria *Lactobacillus rhamnosus* JCM 1136. Veterinary immunology and immunopathology, 102(4), 379-388. |
|  | ***L. salivarius*** | - Aiba, Y., Suzuki, N., Kabir, A.M., Takagi, A. and Koga, Y., 1998. Lactic acid-mediated suppression of *Helicobacter pylori* by the oral administration of *Lactobacillus salivarius* as a probiotic in a gnotobiotic murine model. The American journal of gastroenterology, 93(11), 2097-2101. - Messaoudi, S., Manai, M., Kergourlay, G., Prévost, H., Connil, N., Chobert, J.M. and Dousset, X., 2013. *Lactobacillus salivarius*: bacteriocin and probiotic activity. Food microbiology, 36(2), 296-304. - Peran, L., Camuesco, D., Comalada, M., Nieto, A., Concha, A., Diaz-Ropero, M.P., Olivares, M., Xaus, J., Zarzuelo, A. and Galvez, J., 2005. Preventative effects of a probiotic, *Lactobacillus salivarius* ssp. salivarius, in the TNBS model of rat colitis. World journal of gastroenterology: WJG, 11(33), p.5185. |
| ***Bacillus*** | ***B. Clausii*** | - Urdaci, M.C., Bressollier, P. and Pinchuk, I., 2004. *Bacillus clausii* probiotic strains: antimicrobial and immunomodulatory activities. Journal of clinical gastroenterology, 38, S86-S90. - Lippolis, R., Siciliano, R.A., Mazzeo, M.F., Abbrescia, A., Gnoni, A., Sardanelli, A.M. and Papa, S., 2013. Comparative secretome analysis of four isogenic *Bacillus clausii* probiotic strains. Proteome science, 11(1), p.28. - Bozdogan, B., Galopin, S. and Leclercq, R., 2004. Characterization of a new erm-related macrolide resistance gene present in probiotic strains of *Bacillus clausii.* Applied and environmental microbiology, 70(1), 280-284. - Galopin, S., Cattoir, V. and Leclercq, R., 2009. A chromosomal chloramphenicol acetyltransferase determinant from a probiotic strain of *Bacillus clausii.* FEMS microbiology letters, 296(2), 185-189. |
|  | ***B. circulans*** | - Bandyopadhyay, P. and Mohapatra, P.K.D., 2009. Effect of a probiotic bacterium *Bacillus circulans* PB7 in the formulated diets: on growth, nutritional quality and immunity of *Catla catla* (Ham.). Fish physiology and biochemistry, 35(3), 467-478. - Cavazzoni, V., Adami, A. and Castrovilli, C., 1998. Performance of broiler chickens supplemented with *Bacillus coagulans* as probiotic. British poultry science, 39(4), 526-529. - Balcázar, J.L. and Rojas-Luna, T., 2007. Inhibitory activity of probiotic *Bacillus subtilis* UTM 126 against Vibrio species confers protection against vibriosis in juvenile shrimp (*Litopenaeus vannamei*). Current microbiology, 55(5), 409-412. |
|  | ***B. subtilis*** | - Hong, H.A., Huang, J.M., Khaneja, R., Hiep, L.V., Urdaci, M.C. and Cutting, S.M., 2008. The safety of *Bacillus subtilis* and *Bacillus indicus* as food probiotics. Journal of applied microbiology, 105(2), 510-520. - Aly, S.M., Ahmed, Y.A.G., Ghareeb, A.A.A. and Mohamed, M.F., 2008. Studies on *Bacillus subtilis* and *Lactobacillus acidophilus*, as potential probiotics, on the immune response and resistance of Tilapia nilotica (*Oreochromis niloticus*) to challenge infections. Fish & shellfish immunology, 25(1-2), 128-136. - Williams, P., 2007. *Bacillus subtilis*: a shocking message from a probiotic. Cell Host & Microbe, 1(4), 248-249. - Goya, M.E., Xue, F., Sampedro-Torres-Quevedo, C., Arnaouteli, S., Riquelme-Dominguez, L., Romanowski, A., Brydon, J., Ball, K.L., Stanley-Wall, N.R. and Doitsidou, M., 2020. Probiotic *Bacillus subtilis* Protects against α-Synuclein Aggregation in *C. elegans*. Cell Reports, 30(2), 367-380. - Starosila, D., Rybalko, S., Varbanetz, L., Ivanskaya, N. and Sorokulova, I., 2017. Anti-influenza activity of a *Bacillus subtilis* probiotic strain. Antimicrobial Agents and Chemotherapy, 61(7). - Lefevre, M., Racedo, S.M., Denayrolles, M., Ripert, G., Desfougères, T., Lobach, A.R., Simon, R., Pélerin, F., Jüsten, P. and Urdaci, M.C., 2017. Safety assessment of *Bacillus subtilis* CU1 for use as a probiotic in humans. Regulatory Toxicology and Pharmacology, 83, 54-65. |
|  | ***B. coagulans*** | - Cavazzoni, V., Adami, A. and Castrovilli, C., 1998. Performance of broiler chickens supplemented with *Bacillus coagulans* as probiotic. British poultry science, 39(4), 526-529. - Endres, J.R., Clewell, A., Jade, K.A., Farber, T., Hauswirth, J. and Schauss, A.G., 2009. Safety assessment of a proprietary preparation of a novel Probiotic, *Bacillus coagulans*, as a food ingredient. Food and Chemical Toxicology, 47(6), 1231-1238. - Cao, J., Yu, Z., Liu, W., Zhao, J., Zhang, H., Zhai, Q. and Chen, W., 2020. Probiotic characteristics of *Bacillus coagulans* and associated implications for human health and diseases. Journal of Functional Foods, 64, p.103643. |
|  | ***B. cereus*** | - Li, S.P., Zhao, X.J. and Wang, J.Y., 2009. Synergy of *Astragalus* polysaccharides and probiotics (*Lactobacillus* and *Bacillus cereus*) on immunity and intestinal microbiota in chicks. Poultry science, 88(3), 519-525. - Scharek, L., Altherr, B.J., Tölke, C. and Schmidt, M.F.G., 2007. Influence of the probiotic *Bacillus cereus* var. toyoi on the intestinal immunity of piglets. Veterinary immunology and immunopathology, 120(3-4), 136-147. - Zhu, K., Hölzel, C.S., Cui, Y., Mayer, R., Wang, Y., Dietrich, R., Didier, A., Bassitta, R., Märtlbauer, E. and Ding, S., 2016. Probiotic *Bacillus cereus* strains, a potential risk for public health in China. Frontiers in Microbiology, 7, p.718. - Celandroni, F., Vecchione, A., Cara, A., Mazzantini, D., Lupetti, A. and Ghelardi, E., 2019. Identification of Bacillus species: Implication on the quality of probiotic formulations. PloS one, 14(5), p.e0217021. |
|  | ***B. megaterium*** | - Aftabuddin, S., Kashem, M.A., Kader, M.A., Sikder, M.N.A. and Hakim, M.A., 2013. Use of *Streptomyces fradiae* and *Bacillus megaterium* as probiotics in the experimental culture of tiger shrimp Penaeus monodon (Crustacea, Penaeidae). Aquaculture, Aquarium, Conservation & Legislation, 6(3), 253-267. - Afrilasari, W. and Meryandini, A., 2016. Effect of probiotic *Bacillus megaterium* PTB 1.4 on the population of intestinal microflora, digestive enzyme activity and the growth of catfish (Clarias sp.). HAYATI Journal of Biosciences, 23(4), 168-172. |
|  | ***B. mycoides*** | - Elshaghabee, F.M., Rokana, N., Gulhane, R.D., Sharma, C. and Panwar, H., 2017. Bacillus as potential probiotics: status, concerns, and future perspectives. Frontiers in microbiology, 8, p.1490. |
|  | ***B. pumilus*** | - Yang, H.L., Xia, H.Q., Ye, Y.D., Zou, W.C. and Sun, Y.Z., 2014. Probiotic *Bacillus pumilus* SE5 shapes the intestinal microbiota and mucosal immunity in grouper Epinephelus coioides. Diseases of aquatic organisms, 111(2), 119-127. - Gao, X.Y., Liu, Y., Miao, L.L., Li, E.W., Hou, T.T. and Liu, Z.P., 2017. Mechanism of anti-Vibrio activity of marine probiotic strain *Bacillus pumilus* H2, and characterization of the active substance. AMB Express, 7(1), 1-10. |
|  | ***B. licheniformis*** | - Deng, W., Dong, X.F., Tong, J.M. and Zhang, Q., 2012. The probiotic *Bacillus licheniformis* ameliorates heat stress-induced impairment of egg production, gut morphology, and intestinal mucosal immunity in laying hens. Poultry science, 91(3), 575-582. - Alexopoulos, C., Georgoulakis, I.E., Tzivara, A., Kritas, S.K., Siochu, A. and Kyriakis, S.C., 2004. Field evaluation of the efficacy of a probiotic containing *Bacillus licheniformis* and *Bacillus subtilis* spores, on the health status and performance of sows and their litters. Journal of animal physiology and animal nutrition, 88(11‐12), 381-392. |
| ***Pediococcus*** | ***P. pentosaceus*** | - Vidhyasagar, V. and Jeevaratnam, K., 2013. Evaluation of *Pediococcus pentosaceus* strains isolated from Idly batter for probiotic properties in vitro. Journal of Functional Foods, 5(1), 235-243. - Jonganurakkun, B., Wang, Q., Xu, S.H., Tada, Y., Minamida, K., Yasokawa, D., Sugi, M., Hara, H. and Asano, K., 2008. *Pediococcus pentosaceus* NB-17 for probiotic use. Journal of bioscience and bioengineering, 106(1), 69-73. - Osmanagaoglu, O., Kiran, F. and Ataoglu, H., 2010. Evaluation of in vitro probiotic potential of *Pediococcus pentosaceus* OZF isolated from human breast milk. Probiotics and antimicrobial proteins, 2(3), 162-174. |
|  | ***P. acidilactici*** | - Standen, B.T., Rawling, M.D., Davies, S.J., Castex, M., Foey, A., Gioacchini, G., Carnevali, O. and Merrifield, D.L., 2013. Probiotic *Pediococcus acidilactici* modulates both localised intestinal-and peripheral-immunity in tilapia (*Oreochromis niloticus*). Fish & shellfish immunology, 35(4), 1097-1104. - Castex, M., Lemaire, P., Wabete, N. and Chim, L., 2010. Effect of probiotic *Pediococcus acidilactici* on antioxidant defences and oxidative stress of Litopenaeus stylirostris under Vibrio nigripulchritudo challenge. Fish & shellfish immunology, 28(4), 622-631. |
|  | ***P. ethanolidurans*** | - Iuchi, A., Haruguchi, S., Mongkolthanaruk, W., Arima, J., Nagase, M., Khanh, H.Q., Ichiyanagi, T., Yamaguchi, T., Shimomura, N. and Aimi, T., 2012. Characterization of novel amylase from amylolytic lactic acid bacteria *Pediococcus ethanolidurans* isolated from Japanese pickles (nuka-zuke). *Food Science and Technology Research*, *18*(6), 861-867. |
| ***Vibrio*** | ***V. mediterranei*** | - Thompson, F.L., Gevers, D., Thompson, C.C., Dawyndt, P., Naser, S., Hoste, B., Munn, C.B. and Swings, J., 2005. Phylogeny and molecular identification of vibrios on the basis of multilocus sequence analysis. *Applied and environmental microbiology*, *71*(9), 5107-5115. |
|  | ***V. fluvialis*** | - Sorroza, L., Padilla, D., Acosta, F., Román, L., Grasso, V., Vega, J. and Real, F., 2012. Characterization of the probiotic strain *Vagococcus fluvialis* in the protection of European sea bass (*Dicentrarchus labrax*) against vibriosis by *Vibrio anguillarum*. Veterinary microbiology, 155(2-4), 369-373. |
|  | ***V. gazogenes*** | - Gummadidala, P.M., Chen, Y.P., Beauchesne, K.R., Miller, K.P., Mitra, C., Banaszek, N., Velez-Martinez, M., Moeller, P.D., Ferry, J.L., Decho, A.W. and Chanda, A., 2016. Aflatoxin-exposure of *Vibrio gazogenes* as a novel system for the generation of aflatoxin synthesis inhibitors. Frontiers in microbiology, 7, p.814. |
|  | ***V. alginolyticus*** | - Villamil, L., Figueras, A., Planas, M. and Novoa, B., 2003. Control of *Vibrio alginolyticus* in Artemia culture by treatment with bacterial probiotics. Aquaculture, 219(1-4), 43-56. - Gomez-Gil, B., Roque, A. and Velasco-Blanco, G., 2002. Culture of *Vibrio alginolyticus* C7b, a potential probiotic bacterium, with the microalga Chaetoceros muelleri. Aquaculture, 211(1-4), 43-48. - Ajitha, S., Sridhar, M., Sridhar, N., Bright Singh, I.S. and Varghese, V., 2004. Probiotic effects of lactic acid bacteria against *Vibrio alginolyticus* in Penaeus (Fenneropenaeus) indicus (H. Milne Edwards). Asian Fisheries Science, 17, 71-80. |
| ***Roseobacter*** | ***R. litoralis*** | - Ruiz-Ponte, C., Cilia, V., Lambert, C. and Nicolas, J.L., 1998. *Roseobacter gallaeciensis* sp. nov., a new marine bacterium isolated from rearings and collectors of the scallop Pecten maximus. International Journal of Systematic and Evolutionary Microbiology, 48(2), 537-542. - Piekarski, T., Buchholz, I., Drepper, T., Schobert, M., Wagner-Doebler, I., Tielen, P. and Jahn, D., 2009. Genetic tools for the investigation of *Roseobacter clade* bacteria. BMC microbiology, 9(1), p.265. |
|  | ***R. denitrificans*** | - Piekarski, T., Buchholz, I., Drepper, T., Schobert, M., Wagner-Doebler, I., Tielen, P. and Jahn, D., 2009. Genetic tools for the investigation of *Roseobacter clade* bacteria. BMC microbiology, 9(1), p.265. |
|  | ***R. litoralis 149*** | - Petersen, J., Brinkmann, H., Bunk, B., Michael, V., Päuker, O. and Pradella, S., 2012. Think pink: photosynthesis, plasmids and the *Roseobacter clade*. *Environmental microbiology*, *14*(10), 2661-2672. |
|  | ***R. denitrificans 114*** | - Piekarski, T., Buchholz, I., Drepper, T., Schobert, M., Wagner-Doebler, I., Tielen, P. and Jahn, D., 2009. Genetic tools for the investigation of *Roseobacter clade* bacteria. *BMC microbiology*, *9*(1), 265. |
| ***Vagococcus*** | ***V. fluvialis bH819*** | - Fu, S., Xia, W., Wang, Q., Rahman, M.M., Hao, J., Ye, S., Liu, Y. and Li, R., 2020. Genomic characterization and pathogenicity analysis of the probiotic *Vagococcus lutrae* strain VL-18 causing severe skin lesions in warm-blooded animals. Aquaculture, p.735166. |
|  | ***V. teuberi*** | - Fu, S., Xia, W., Wang, Q., Rahman, M.M., Hao, J., Ye, S., Liu, Y. and Li, R., 2020. Genomic characterization and pathogenicity analysis of the probiotic *Vagococcus lutrae* strain VL-18 causing severe skin lesions in warm-blooded animals. Aquaculture, p.735166. |
| ***Oenococcus*** | ***O. oeni*** | - Foligné, B., Dewulf, J., Breton, J., Claisse, O., Lonvaud-Funel, A. and Pot, B., 2010. Probiotic properties of non-conventional lactic acid bacteria: immunomodulation by *Oenococcus oeni*. International journal of food microbiology, 140(2-3), 136-145. |
|  | ***O. kitaharae*** | - Zanirati, D.F., Abatemarco Jr, M., de Cicco Sandes, S.H., Nicoli, J.R., Nunes, Á.C. and Neumann, E., 2015. Selection of lactic acid bacteria from Brazilian kefir grains for potential use as starter or probiotic cultures. Anaerobe, 32, 70-76. |
|  | ***O. alcoholitolerans*** | - Badotti, F., Moreira, A.P.B., Tonon, L.A.C., de Lucena, B.T.L., Fátima de Cássia, O.G., Kruger, R., Thompson, C.C., de Morais, M.A., Rosa, C.A. and Thompson, F.L., 2014. *Oenococcus alcoholitolerans* sp. nov., a lactic acid bacteria isolated from cachaça and ethanol fermentation processes. Antonie van Leeuwenhoek, 106(6), 1259-1267. |
|  | ***O. oeni AWRIB429*** | - Favier, M., Bilhère, E., Lonvaud-Funel, A., Moine, V. and Lucas, P.M., 2012. Identification of pOENI-1 and related plasmids in Oenococcus oeni strains performing the malolactic fermentation in wine. PLoS One, 7(11), p.e49082. |
| ***Pseudomonas*** | ***P. fluorescens*** | - Gram, L., Melchiorsen, J., Spanggaard, B., Huber, I. and Nielsen, T.F., 1999. Inhibition of *Vibrio anguillarum* by *Pseudomonas fluorescens* AH2, a possible probiotic treatment of fish. Applied and environmental microbiology, 65(3), 969-973. - Eissa, N., Abou El-Gheit, N. and Shaheen, A.A., 2014. Protective effect of *Pseudomonas fluorescens* as a probiotic in controlling fish pathogens. American Journal of BioScience, 2(5), 175-181. |
|  | ***P. chlororaphis*** | - Anderson, A.J. and Kim, Y.C., 2018. Biopesticides produced by plant-probiotic *Pseudomonas chlororaphis* isolates. Crop Protection, 105, pp.62-69. |
|  | ***P. stutzeri*** | - Feliatra, F., Muchlisin, Z.A., Teruna, H.Y., Utamy, W.R., Nursyirwani, N. and Dahliaty, A., 2018. Potential of bacteriocins produced by probiotic bacteria isolated from tiger shrimp and prawns as antibacterial to *Vibrio*, *Pseudomonas*, and *Aeromonas* species on fish. F1000Research, 7. - Abdelkarim, M., Kamel, C., Fathi, K. and Amina, B., 2010. Use of *Pseudomonas stutzeri* and *Candida utilis* in the improvement of the conditions of Artemia culture and protection against pathogens. Brazilian Journal of Microbiology, 41(1), 107-115. |
|  | ***P. synxantha*** | - Hai, N.V., Buller, N. and Fotedar, R., 2009. Effects of probiotics (*Pseudomonas synxantha* and *Pseudomonas aeruginosa*) on the growth, survival and immune parameters of juvenile western king prawns (*Penaeus latisulcatus* Kishinouye, 1896). Aquaculture Research, 40(5), 590-602. |
| ***Shewanella*** | ***S. colwelliana*** | - Jiang, H.F., Liu, X.L., Chang, Y.Q., Liu, M.T. and Wang, G.X., 2013. Effects of dietary supplementation of probiotic *Shewanella colwelliana* WA64, *Shewanella olleyana* WA65 on the innate immunity and disease resistance of abalone, Haliotis discus hannai Ino. Fish & shellfish immunology, 35(1), 86-91. |
|  | ***S. putrefaciens*** | - Lobo, C., Moreno-Ventas, X., Tapia-Paniagua, S., Rodríguez, C., Morinigo, M.A. and de La Banda, I.G., 2014. Dietary probiotic supplementation (*Shewanella putrefaciens* Pdp11) modulates gut microbiota and promotes growth and condition in Senegalese sole larviculture. Fish physiology and biochemistry, 40(1), 295-309. - Tapia-Paniagua, S.T., Vidal, S., Lobo, C., Prieto-Álamo, M.J., Jurado, J., Cordero, H., Cerezuela, R., de la Banda, I.G., Esteban, M.A., Balebona, M.C. and Moriñigo, M.A., 2014. The treatment with the probiotic *Shewanella putrefaciens* Pdp11 of specimens of *Solea senegalensis* exposed to high stocking densities to enhance their resistance to disease. Fish & shellfish immunology, 41(2), 209-221. |
|  | ***S. xiamenensis*** | - Hao, K., Wu, Z.Q., Li, D.L., Yu, X.B., Wang, G.X. and Ling, F., 2017. Effects of dietary administration of *Shewanella xiamenensis* A-1, *Aeromonas veronii* A-7, and *Bacillus subtilis*, single or combined, on the grass carp (*Ctenopharyngodon idella*) intestinal microbiota. Probiotics and antimicrobial proteins, 9(4), 386-396. - Wu, Z.Q., Jiang, C., Ling, F. and Wang, G.X., 2015. Effects of dietary supplementation of intestinal autochthonous bacteria on the innate immunity and disease resistance of grass carp (*Ctenopharyngodon idellus*). Aquaculture, 438, 105-114. |
| ***Enterococcus*** | ***E. durans*** | - Pieniz, S., Andreazza, R., Anghinoni, T., Camargo, F. and Brandelli, A., 2014. Probiotic potential, antimicrobial and antioxidant activities of *Enterococcus durans* strain LAB18s. Food Control, 37, 251-256. - Pieniz, S., de Moura, T.M., Cassenego, A.P.V., Andreazza, R., Frazzon, A.P.G., de Oliveira Camargo, F.A. and Brandelli, A., 2015. Evaluation of resistance genes and virulence factors in a food isolated *Enterococcus durans* with potential probiotic effect. Food Control, 51, 49-54. |
|  | ***E. faecium*** | - Scharek, L., Guth, J., Reiter, K., Weyrauch, K.D., Taras, D., Schwerk, P., Schierack, P., Schmidt, M.F.G., Wieler, L.H. and Tedin, K., 2005. Influence of a probiotic *Enterococcus faecium* strain on development of the immune system of sows and piglets. Veterinary immunology and immunopathology, 105(1-2), 151-161. - Taras, D., Vahjen, W., Macha, M. and Simon, O., 2006. Performance, diarrhea incidence, and occurrence of Escherichia coli virulence genes during long-term administration of a probiotic *Enterococcus faecium* strain to sows and piglets. Journal of animal science, 84(3), 608-617. |
|  | ***E. faecalis*** | - Nueno-Palop, C. and Narbad, A., 2011. Probiotic assessment of *Enterococcus faecalis* CP58 isolated from human gut. International journal of food microbiology, 145(2-3), 390-394. - Domann, E., Hain, T., Ghai, R., Billion, A., Kuenne, C., Zimmermann, K. and Chakraborty, T., 2007. Comparative genomic analysis for the presence of potential enterococcal virulence factors in the probiotic *Enterococcus faecalis* strain *Symbioflor* 1. International Journal of Medical Microbiology, 297(7-8), 533-539. |
|  | ***E. raffinosus*** | - Peixoto, M.J., Domingues, A., Batista, S., Gonçalves, J.F.M., Gomes, A.M., Cunha, S., Valente, L.M.P., Costas, B. and Ozório, R.O.A., 2018. Physiopathological responses of sole (*Solea senegalensis*) subjected to bacterial infection and handling stress after probiotic treatment with autochthonous bacteria. Fish & shellfish immunology, 83, 348-358. |
|  | ***E. hirae*** | - Gupta, A. and Tiwari, S.K., 2015. Probiotic potential of bacteriocin-producing *Enterococcus hirae* strain LD3 isolated from dosa batter. Annals of microbiology, 65(4), 2333-2342. - Arokiyaraj, S., Islam, V.I.H., Bharanidharan, R., Raveendar, S., Lee, J., Kim, D.H., Oh, Y.K., Kim, E.K. and Kim, K.H., 2014. Antibacterial, anti-inflammatory and probiotic potential of *Enterococcus hirae* isolated from the rumen of Bos primigenius. World Journal of Microbiology and Biotechnology, 30(7), 2111-2118. |
|  | ***E. mundtii*** | - Botes, M., van Reenen, C.A. and Dicks, L.M., 2008. Evaluation of *Enterococcus mundtii* ST4SA and Lactobacillus plantarum 423 as probiotics by using a gastro-intestinal model with infant milk formulations as substrate. International journal of food microbiology, 128(2), 362-370. - Botes, M., Loos, B., van Reenen, C.A. and Dicks, L.M., 2008. Adhesion of the probiotic strains *Enterococcus mundtii* ST4SA and *Lactobacillus plantarum* 423 to Caco-2 cells under conditions simulating the intestinal tract, and in the presence of antibiotics and anti-inflammatory medicaments. Archives of microbiology, 190(5), 573-584. |
| ***Bifidobacterium*** | ***B. animalis*** | - Jungersen, M., Wind, A., Johansen, E., Christensen, J.E., Stuer-Lauridsen, B. and Eskesen, D., 2014. The Science behind the Probiotic Strain *Bifidobacterium animalis* subsp. lactis BB-12®. Microorganisms, 2(2), 92-110. - Kim, J.F., Jeong, H., Yu, D.S., Choi, S.H., Hur, C.G., Park, M.S., Yoon, S.H., Kim, D.W., Ji, G.E., Park, H.S. and Oh, T.K., 2009. Genome sequence of the probiotic bacterium *Bifidobacterium animalis* subsp. lactis AD011. Journal of bacteriology, 191(2), 678-679. |
|  | ***B. bifidum*** | - Hekmat, S. and McMAHON, D.J., 1992. Survival of *Lactobacillus acidophilus* and *Bifidobacterium bifidum* in ice cream for use as a probiotic food. Journal of dairy science, 75(6), 1415-1422. - Hernandez-Mendoza, A., Robles, V.J., Angulo, J.O., De La Cruz, J. and Garcia, H.S., 2007. Preparation of a whey-based probiotic product with *Lactobacillus reuteri* and *Bifidobacterium bifidum*. Food Technology and Biotechnology, 45(1), 27-31. |
|  | ***B. longum*** | - Messaoudi, M., Lalonde, R., Violle, N., Javelot, H., Desor, D., Nejdi, A., Bisson, J.F., Rougeot, C., Pichelin, M., Cazaubiel, M. and Cazaubiel, J.M., 2011. Assessment of psychotropic-like properties of a probiotic formulation (*Lactobacillus helveticus* R0052 and *Bifidobacterium longum* R0175) in rats and human subjects. British Journal of Nutrition, 105(5), 755-764. - Messaoudi, M., Violle, N., Bisson, J.F., Desor, D., Javelot, H. and Rougeot, C., 2011. Beneficial psychological effects of a probiotic formulation (*Lactobacillus helveticus* R0052 and *Bifidobacterium longum* R0175) in healthy human volunteers. Gut microbes, 2(4), 256-261. - Pinto-Sanchez, M.I., Hall, G.B., Ghajar, K., Nardelli, A., Bolino, C., Lau, J.T., Martin, F.P., Cominetti, O., Welsh, C., Rieder, A. and Traynor, J., 2017. Probiotic *Bifidobacterium longum* NCC3001 reduces depression scores and alters brain activity: a pilot study in patients with irritable bowel syndrome. Gastroenterology, 153(2), 448-459. |
|  | ***B. breve*** | - Jeon, S.G., Kayama, H., Ueda, Y., Takahashi, T., Asahara, T., Tsuji, H., Tsuji, N.M., Kiyono, H., Ma, J.S., Kusu, T. and Okumura, R., 2012. Probiotic *Bifidobacterium breve* induces IL-10-producing Tr1 cells in the colon. PLoS Pathog, 8(5), p.e1002714. - Shimakawa, Y., Matsubara, S., Yuki, N., Ikeda, M. and Ishikawa, F., 2003. Evaluation of *Bifidobacterium breve* strain Yakult-fermented soymilk as a probiotic food. International journal of food microbiology, 81(2), 131-136. |
|  | ***B. adolescentis*** | - Annan, N.T., Borza, A.D. and Hansen, L.T., 2008. Encapsulation in alginate-coated gelatin microspheres improves survival of the probiotic *Bifidobacterium adolescentis* 15703T during exposure to simulated gastro-intestinal conditions. Food Research International, 41(2), 184-193. - Cole, C.B., Fuller, R. and Carter, S.M., 1989. Effect of probiotic supplements of lactobacillus acidophilus and *Bifidobacterium adolescentis* 2204 on β-glueosidase and β-glueuronidase activity in the lower gut of rats associated with a human faecal flora. Microbial Ecology in Health and Disease, 2(3), 223-225. |
| Carnobacterium | ***C. divergens*** | - Kim, D.H. and Austin, B., 2006. Innate immune responses in rainbow trout (*Oncorhynchus mykiss*, Walbaum) induced by probiotics. Fish & shellfish immunology, 21(5), 513-524. - Gildberg, A., Mikkelsen, H., Sandaker, E. and Ringø, E., 1997. Probiotic effect of lactic acid bacteria in the feed on growth and survival of fry of Atlantic cod (*Gadus morhua*). Hydrobiologia, 352(1-3), 279-285. |
|  | ***C.maltaromaticum*** | - Leisner, J.J., Hansen, M.A., Larsen, M.H., Hansen, L., Ingmer, H. and Sørensen, S.J., 2012. The genome sequence of the lactic acid bacterium, *Carnobacterium maltaromaticum* ATCC 35586 encodes potential virulence factors. International journal of food microbiology, 152(3), 107-115. - Løvmo Martinsen, L., Salma, W., Myklebust, R., Mayhew, T.M. and Ringø, E., 2011. *Carnobacterium maltaromaticum* vs. Vibrio (Listonella) anguillarum in the midgut of Atlantic cod (*Gadus morhua* L.): an ex vivo study. Aquaculture Research, 42(12), 1830-1839. |
| Lactococcus | ***L. lactis*** | - Desmond, C., Fitzgerald, G.F., Stanton, C. and Ross, R.P., 2004. Improved stress tolerance of GroESL-overproducing *Lactococcus lactis* and probiotic *Lactobacillus paracasei* NFBC 338. Applied and environmental microbiology, 70(10), 5929-5936. - Todorov, S.D., Botes, M., Danova, S.T. and Dicks, L.M.T., 2007. Probiotic properties of *Lactococcus lactis* ssp. lactis HV219, isolated from human vaginal secretions. Journal of Applied Microbiology, 103(3), 629-639. |
| Leuconostock | ***L. mesenteroides*** | - Kekkonen, R.A., Kajasto, E., Miettinen, M., Veckman, V., Korpela, R. and Julkunen, I., 2008. Probiotic *Leuconostoc mesenteroides* ssp. cremoris and *Streptococcus thermophilus* induce IL-12 and IFN-γ production. World journal of gastroenterology: WJG, 14(8), 1192. - Beganović, J., Pavunc, A.L., Gjuračić, K., Špoljarec, M., Šušković, J. and Kos, B., 2011. Improved sauerkraut production with probiotic strain *Lactobacillus plantarum* L4 and *Leuconostoc mesenteroides* LMG 7954. Journal of Food Science, 76(2), M124-M129. |
| Micrococcus | ***M. luteus*** | - Abd El-Rhman, A.M., Khattab, Y.A. and Shalaby, A.M., 2009. *Micrococcus luteus* and Pseudomonas species as probiotics for promoting the growth performance and health of Nile tilapia, *Oreochromis niloticus*. Fish & Shellfish Immunology, 27(2), 175-180. |
| Streptococcus | ***S. salivarius*** | - Burton, J.P., Chilcott, C.N. and Tagg, J.R., 2005. The rationale and potential for the reduction of oral malodour using *Streptococcus salivarius* probiotics. Oral diseases, 11, 29-31. - Wescombe, P.A., Hale, J.D., Heng, N.C. and Tagg, J.R., 2012. Developing oral probiotics from *Streptococcus salivarius*. Future microbiology, 7(12), 1355-1371. |
|  | ***S. thermophilus*** | - Kekkonen, R.A., Kajasto, E., Miettinen, M., Veckman, V., Korpela, R. and Julkunen, I., 2008. Probiotic *Leuconostoc mesenteroides* ssp. cremoris and *Streptococcus thermophilus* induce IL-12 and IFN-γ production. World journal of gastroenterology: WJG, 14(8), 1192. - Champagne, C.P., Green-Johnson, J., Raymond, Y., Barrette, J. and Buckley, N., 2009. Selection of probiotic bacteria for the fermentation of a soy beverage in combination with *Streptococcus thermophilus*. Food Research International, 42(5-6), 612-621. |
| Paenibacillus | ***P. polymyxa*** | - Midhun, S.J., Neethu, S., Vysakh, A., Arun, D., Radhakrishnan, E.K. and Jyothis, M., 2017. Antibacterial activity and probiotic characterization of autochthonous *Paenibacillus polymyxa* isolated from *Anabas testudineus* (Bloch, 1792). Microbial pathogenesis, 113, 403-411. - Naghmouchi, K., Baah, J., Cudennec, B. and Drider, D., 2013. Required characteristics of *Paenibacillus polymyxa* JB-0501 as potential probiotic. Archives of microbiology, 195(8), 537-543. |
| Aeromonas | ***A. veronii*** | - Hao, K., Wu, Z.Q., Li, D.L., Yu, X.B., Wang, G.X. and Ling, F., 2017. Effects of dietary administration of *Shewanella xiamenensis* A-1, *Aeromonas veronii* A-7, and Bacillus subtilis, single or combined, on the grass carp (*Ctenopharyngodon idella*) intestinal microbiota. Probiotics and antimicrobial proteins, 9(4), 386-396. |
