# Supplementary material for: Metagenome analysis from the sediment of river Ganga and Yamuna: In search of beneficial microbiome
Source: PLoS One. 2020 Oct 6;15(10):e0239594. doi: 10.1371/journal.pone.0239594 (PMC7537857; doi:10.1371/journal.pone.0239594)
Supplement: Attachment — Submitted filename: Response to Reviewers.docx [file pone.0239594.s003.docx]

**Reply to Reviewers Comment**

| **Sl. No.** | **General Comment** | **Response to General Comment** |
| --- | --- | --- |
| 1. | We note that Figure 1 in your submission contain map/satellite images which may be copyrighted. All PLOS content is published under the Creative Commons Attribution License (CC BY 4.0), which means that the manuscript, images, and Supporting Information files will be freely available online, and any third party is permitted to access, download, copy, distribute, and use these materials in any way, even commercially, with proper attribution. For these reasons, we cannot publish previously copyrighted maps or satellite images created using proprietary data, such as Google software (Google Maps, Street View, and Earth). For more information, see our copyright guidelines:  <http://journals.plos.org/plosone/s/licenses-and-copyright>.  1) Thank you for letting us know the Map in Figure 1 was created used ArcGIS 10.2.1 platform.  Before we proceed, please also clarify where the data used in the map is from. If the authors used their own data for the map, please let us know. | Thanks for suggestion.  As per the suggestion, the Figure 1 has been modified using ArcGIS 10.2.1 platform.  The data used in preparation of the map was own data of the authors. |

| **Reviewer # 1** | | |
| --- | --- | --- |
| **Sl. No.** | **Reviewer’s Comments** | **Response to Reviewer’s Comments** |
| **Major Comments** | | |
| 1. | The criteria used for construction of the beneficial bacteria list are incomprehensible. It looks very strange that beneficial effects of each of these 69 bacteria (Supplementary Table 1) were evidenced by only one study. A simple pubmed search indicates that several of them have pathogenic effect to aquatic organisms. For example, *Vibrio mediterranei* is associated to major mortality in *Pinna nobilis*(https://www.sciencedirect.com/science/article/abs/pii/S0044848619324494). Some have role in serious human health hazards. For example consumption of fish infected with *V. fluvialis* has been reported to cause mild to moderate dehydration, vomiting, fever, abdominal pain and diarrhoea(https://www.ncbi.nlm.nih.gov/pmc/articles/PMC2996184/). There are many more such bacteria from this list which has been reported to be associated with serious illness in human and other aquatic organisms. So authors need to define clear criteria for this classification and provide more than one evidence supporting their health benefit. | The beneficial effects of the identified bacteria from the metagenome sequence data were verified again with more literatures to confirm. Study revealed that, few strains of the identified bacterial species are beneficial in nature; however same bacterial species are also pathogenic which may be due to different strains of same species. The pathogenic activities of the same bacterial species may be due to the variations in genome. They may be representing different ecotype or biotype. The details have been provided as Supplementary Table S1 and S2. |
| 2. | The abstract describes that a total of 242 health beneficial bacteria were identified. However, rest of the manuscript discusses only 69 (Heatmap figure legend says 67 species). While Kaiju web server performs taxonomic classification of sequencing reads, it is unclear from the manuscript whether the list of beneficial bacteria was created before performing the all data analysis or bacteria names (form Kaiju results) were selected later after a manual literature search? | The MS has been modified. The 69 helpful bacteria have been reported in this MS instead of 242. Heat map figure legend has been changed accordingly. The list of bacteria names (form Kaiju results) were selected after a manual literature search. |
| 3. | The method section is ambiguous and insufficient to reproduce the results independently. Important analyses such as relative abundance calculation and statistical methods used for significance tests have not been described sufficiently. | Thanks for suggestion.  The identified beneficial bacteria found in our metagenomic data were manually checked with NCBI references for conformation. Relative abundance of beneficial bacteria was calculated using Kaiju Web Server. Comparison was done based on standard student t-test following the Reference Imchen et al., 2018. They did similar kind of metagenomic research. |
| 4. | In many cases the input data are not clear. For example, the inputs for multiple sequence analysis (or multiple Sequence alignment???), species Phylogeny construction and PCA are not clear from the text. | The Phylogenetic tree has been reconstructed and PCA analysis has been revised. |
| 5. | The purpose of Phylogenetic analysis should be discussed appropriately. The authors conclude that "some of the microbial species are highly conserved throughout the evolution". How come a species can be conserved? Genes/proteins are conserved during evolution of a lineage.  - Bootstrap is a score to show confidence of relatedness or similarity in a clade and does not refer to species closeness.  - Pg 15: What is "evolutionary closed supportive microbiome”??  - Pg 20: What is the difference between "Circular Cladogram" and "Cladogram"? | The Phylogenetic tree has been reconstructed considering 69 identified helpful bacteria with the Bootstrap values. The phylogenetics description part in the MS has been modified accordingly. |
| 6. | The Relative abundance calculation is totally unclear. In biodiversity studies usually it refers to the number of individuals from i-th pecies divided by total number of individuals from a sample (10.1590/S1516-89132012000200014). It would be useful if the authors provide a concise definition of this index at species level. | Thanks for suggestion.  Relative abundance was calculated from total number of bacterial population generated from metagenomic sequences. The MS has been modified as suggested by the Reviewer. |
| 7. | The GO enrichment and pathway enrichment does not show any relation with heavy metal re-mediation. Mere presence of HMA domain in one protein from one species should not be concluded as "potential heavy metal remediating property".  This result should be reported in a subtle way. | The Functional metagenomics analysis in the Result section of the MS has been removed. The same in the Methods and Discussion part of the MS has also been removed. |
| 8. | The rationale behind the STRING protein-protein interaction is not clear. | STRING protein-protein interaction part has been removed from the MS. |
| 9. | In introduction, the manuscript describes that, there are many studies on discovering microbiome from natural streams of other countries. However, in the discussion the results obtained from present study were not compared enough with results from past metagenomic studies. The discussion should put the results in a more broader context. | As suggested by the reviewer, the results obtained from present study has been compared with results from past metagenomic studies in the discussion. |
| 10. | The conclusion that "sediment metagenome of the river Ganga and Yamuna manifests the enriched microbial distribution of health beneficiary microbes" seems biased. Since the study analyses pre-selected list of 69 species, whether the samples are enriched in beneficial bacteria or not is difficult to conclude. | The 69 species of helpful bacteria were identified from our Metagenome data and confirmed with published literatures about their benefits. The detail information has been provided in the Supplementary Table S1 and S2. |
| **Minor Comments** | | |
| 1. | Lack of page and line numbers in the manuscript makes it hard to review. I have tried to write the exact phrase so that  the authors can find the concerned lines. | Modified the MS as suggested |
| 2. | Reference-1 seems bit out of context. Also the list of authors seems incomplete (Compare with this  https://www.scienceopen.com/document?vid=b20de2d9-e8dd-478e-8e2b-5fb9d967b6ea) | Reference-1 has been removed from the MS |
| 3. | Pg -12 "of health beneficial microbiome" should be "beneficial microbiome" | Modified the MS as suggested |
| 4. | Substitution models and number of bootstrap replicate number should be mentioned in a phylogenetic analysis. | The bootstrap replicate number have been mentioned in the phylogenetic tree |
| 5. | For all software tools please provide appropriate citation. Citations help the labs to receive funding and maintain the  tool. | Modified the MS as suggested |
| 6. | Pg-12: "each sample was then assembled into scaffolds using CLC". Please provide the number of scaffolds and their Average length. There is no information about these scaffolds in the results. | Modified the MS as suggested |
| 7. | Pg 14: Please fix the scientific names: *Bacillus Clausii, Enterococcus Hirae, Leuconostocme senteroides, Pediococcus Acidilactici, Pseudomonas Stutzeri, Roseobacter Litoralis* | Corrected the MS as suggested |
| 8. | Pg-14: "four Roseobacter (*R. litoralis, R. denitrificans, R. Litoralis and R. denitrificans*)"- *R. litoralis* and *R. Litoralis* are similar or different. It makes the list of species to 68. | Corrected the MS as suggested |
| 9. | Multiple species has Similar genus abbreviation. Consider using two letter abbreviation; for example Vi. mediterranei  and Va. fluvialis bH819. | Corrected the MS as suggested |
| 10. | "Lactococcuslactis " should be *Lactococcus lactis* ??? | Corrected the MS as suggested |
| 11. | "*Leuconostocme senteroides*" should be *Leuconostoc mesenteroides* ??? | Corrected the MS as suggested |
| 12. | "boot strap" or bootstrap ??? | Corrected the MS as suggested |
| 13. | Pg 15: Heatmap is a way of data representation. What is Heatmap analysis?? | Corrected the MS as suggested |
| 14. | Pg 15: "beneficial species in the" should be "beneficial species in the”?? | Corrected the MS as suggested |
| 15. | The term "significantly" should be used more cautiously. It should be used only for statistical tests and the alpha (pvalue) must be specified. | Modified the MS as suggested |
| 16. | The term "a large number of" seems very vague. Rather, such numbers should be described in a quantitative manner  (in terms of percentage). | Modified the MS as suggested |
| 17. | Pg 35: I would suggest replacing Table 2 with a box-plot. It would reduce the number of pages while making the results more informative and attractive. | Thanks for suggestion  Tabular form in the MS would be more useful |
| 18. | The bootstrap values should be displayed on the tree in appropriate way. | The bootstrap values have been displayed on the tree in appropriate way |

| **Reviewer # 2** | | |
| --- | --- | --- |
| **Sl. No.** | **Reviewer’s Comments** | **Response to Reviewer’s Comments** |
| 1. | In method logic behind setting up string analysis parameter could be mentioned. | The string analysis part has been removed from the MS. |
| 2. | Authors could mention limitations and future direction of the study in the conclusion. | The MS has been modified as suggested |
